# Editorial: Understanding the crosstalk between immune cells and the tumor microenvironment in cancer and its implications for immunotherapy

**DOI:** 10.3389/fmed.2023.1202581

**Published:** 2023-06-06

**Authors:** Noha Mousaad Elemam, Iman Mamdouh Talaat, Reem Amr Assal, Rana A. Youness

**Affiliations:** ^1^Research Institute for Medical and Health Sciences, University of Sharjah, Sharjah, United Arab Emirates; ^2^Department of Clinical Sciences, College of Medicine, University of Sharjah, Sharjah, United Arab Emirates; ^3^Department of Pharmacology and Toxicology, Heliopolis University for Sustainable Development (HU), Cairo, Egypt; ^4^Biology and Biochemistry Department, Molecular Genetics Research Team, School of Life and Medical Sciences, University of Hertfordshire Hosted by Global Academic Foundation, Cairo, Egypt

**Keywords:** colorectal cancer, breast cancer, prognosis, cancer, immune cells, immunotherapy, tumor microenvironment, lung cancer

This editorial features the articles published in this Research Topic in Frontiers in Medicine. This Research Topic aimed to uncover the complex interactions between tumor cells, immune cells, and their microenvironment, as well as their implications in cancer immunotherapy. Also, this topic aimed to provide insights into various crosstalk mechanisms that could be translated into the clinics. A case report by Liu et al. reported a 68-year-old male with chemotherapy-intolerable stage IV intrahepatic cholangiocarcinoma. This study revealed that the biomarkers predicting the response to immunotherapy failed to accurately capture the treatment response and clinical benefit of anti-PD-1 immunotherapy (Liu et al.). Moreover, lung metastasis occurred despite the shrinkage of the primary liver tumor and metastasis in the lymph nodes when anti-PD-1 immunotherapy was combined with radiotherapy. However, with the continued administration of radiotherapy and immunotherapy, a complete response was evident for the primary tumor and metastatic lesions with no treatment-related adverse effects.

Another study discussed another immunotherapeutic approach which is cytokine-based therapy (Razeghian et al.). The toxicity of cytokine-based therapeutics is attributed to the high doses required to reach the anticipated outcome, which limited their clinical utility and led to the employment of mesenchymal stem/stromal cells (MSCs) as potential vehicles for cytokine delivery in various tumors owing to their relatively low immunogenicity and tumor tropism (Razeghian et al.). Despite their unfavorable effects on drug resistance and metastasis, the use of MSC-based cytokine delivery systems can lead to effective immune cell-induced anti-tumor response and provide sustained cytokine release. Current research advances suggest that the combined use of engineered MSCs and small molecules could result in their notable safety and therapeutic efficacy.

The systemic review by Numprasit et al. highlighted the association between the expression of carbonic anhydrase IX (CAIX), a reliable endogenous marker of hypoxia, and BC patients' survival. It was reported that high expression of CAIX was associated with poor disease-free survival (DFS) in 9,157 BC patients. Furthermore, upon classifying BC patients according to their molecular subtypes, high CAIX expression was found to be associated with poor DFS and overall survival (OS) in the triple-negative subtype and a shorter DFS in the hormonal-positive subtype. This indicated that high CAIX expression is a poor prognostic indicator regardless of the subtypes and could be a potential therapeutic target in BC.

Hua et al., in this study, focused on the association between ovarian aging and BC risk. In this research article, the authors performed a multicohort genetic analysis, where clinicopathological data and gene expression data for 3366 BC patients were retrieved and analyzed. The results showed that the eight-validated Ovarian aging-related genes (OARG)-based signature established a prognostic model for BC using independent cohorts. Furthermore, a nomogram with good predictive performance was implemented by incorporating the OARG risk score with the clinicopathological factors. It is also worth noting that the OARG-based signature correlated with DNA damage repair, immune cell signaling pathways, and immunomodulatory functions. Collectively, this study postulated a comprehensive analytical method for BC assessment based on a unique eight OARG signature, which could accurately predict clinical outcomes and drug sensitivity of BC patients.

Decoding genomic and epigenetic changes in tumor cells has helped scientists comprehend the nature of cancer and find curative ways, including the contemporary notion of immunotherapies. The mini-review article by Talaat and Kim discussed the tumor microenvironment (TME) as a compartment guiding the dynamic interplay of different cell types. Also, they reviewed numerous initiatives, such as data-driven strategies, that will quickly advance our knowledge of the environment in which tumor cells thrive, leading to novel findings of prognostic indicators and eventually resulting in overcoming resistance to management.

The TME is known to consist of tumor-infiltrating lymphocytes (TILs), tumor-associated macrophages (TAMs), and tumor-associated neutrophils (TANs). The review by Talaat et al. highlights the several immune checkpoint molecules that are expressed on these immune cells and their interaction with colon cancer cells. Thus, novel approaches for therapy for solid tumors such as colorectal cancer (CRC) are targeting immune checkpoint markers; however, there are still obstacles to successful treatment. On the other hand, the article by Liu and Wang reviewed the use of TAMs in immunotherapy. Whilst macrophages are phagocytic cells that perform a variety of roles in the protection against external invaders, TAMs enhance tumor development and progression by supporting tumor cell division and invasion, immunosuppression, and angiogenesis, which is linked to the poor prognosis in the majority of solid tumors. As a result, an in-depth understanding of TAMs can lead to the discovery of more successful cancer treatment methods. Currently, a significant number of TAM-targeting medicinal drugs are in clinical studies.

The article by Banna et al. explored new techniques for quantitative image analysis, like radiomics or pathomics, which may provide a thorough method for analyzing spatial and temporal data from macroscopic imaging features that may be indicative of underlying molecular drivers and tumor-immune microenvironment in addition to the prognosis after immunotherapy. Additionally, merging data from other sources, such as blood levels, molecular characteristics, radiomics, and pathomics can boost the precision of their models. As a result, “digital biopsy”, as a non-invasive digital method, may have the ability to enable a tailored strategy for cancer patients.

Due to the limitations of immunotherapy in CRC, the review by Mahgoub et al.explores the manipulation of autophagy as a possible adjuvant therapeutic method for patients with different molecular subtypes of CRC. The molecular regulation of autophagy in CRC and how it impacts numerous mechanisms and processes that regulate TME, as well as its role in the development of CRC, tumor immunity, hypoxia, and oxidative stress. Moreover, the clinical efforts and difficulties associated with combining autophagy modulators with other cancer-targeted drugs were discussed to improve CRC patients' survival and slow disease progression.

Rashid et al. reviewed the diagnosis, prognosis, and therapeutic approaches of CRC by shedding light on non-steroidal anti-inflammatory drugs (NSAIDs) that are commonly used as analgesics and anti-inflammatory agents. They have highlighted that NSAIDs possess a potent chemo-preventive effect on several gastrointestinal malignancies, including CRC, in several epidemiological and preclinical studies. The authors also described the molecular mechanisms postulated by which NSAIDs could act as chemo-preventive agents by preventing the synthesis of prostaglandins and resulting in NSAID-induced apoptosis and CRC growth inhibition.

Currently, an increasing number of studies examine the role of RNA modifications such as N7-methylguanosine (m7G) in tumors. A significant m7G-related signature, known as the m7G score, was elucidated based on four principal genes, namely *E2F7, FAM83A, PITX3*, and *HOXA13*, for predicting the immune infiltration and prognosis of lung adenocarcinoma (LUAD) (Li et al.). The m7G score could preferentially differentiate between two distinct molecular subtypes of LUAD. Moreover, the higher m7G score indicated poorer prognosis, higher immune infiltration, significant PD-1 and PD-L1 upregulation, higher tumor mutational burden, and lower tumor immune dysfunction and exclusion scores. Such an approach could aid in the advancement of novel therapeutic strategies for LUAD.

Zajac et al. focused in their review article on MAGE-A antigens, which are the first identified molecular human tumor-associated antigens. The authors shed light on their high tumor specificity and their potential usage as attractive targets for cancer immunotherapies. The review article was mainly focusing on structural features and functional aspects of MAGE-A antigens. Nonetheless, the authors reviewed all past and ongoing clinical studies targeting MAGE-A antigens, as well as the pros and cons of different therapeutic approaches.

## Author contributions

All authors listed have made a substantial, direct, and intellectual contribution to the work and approved it for publication.

